# Aquaporin 11-Dependent Inhibition of Proliferation by Deuterium Oxide in Activated Hepatic Stellate Cells

**DOI:** 10.3390/molecules23123209

**Published:** 2018-12-05

**Authors:** Phil Jun Lee, Hye-Jin Park, Namki Cho, Hong Pyo Kim

**Affiliations:** 1 College of Pharmacy, Ajou University, Suwon 16499, Korea; phil@ajou.ac.kr (P.J.L.); hyejin133@ajou.ac.kr (H.-J.P.); 2 College of Pharmacy and Research Institute of Drug Development, Chonnam National University, Gwangju 61186, Korea

**Keywords:** hepatic stellate cell, deuterium oxide, aquaporin 11, AQP11, heme oxygenase 1, HO-1, bisdemethoxycurcumin

## Abstract

Deuterium oxide (D_2_O) has been reported to be active toward various in vitro cell lines in combination with phytochemicals. Our objective was to describe, for the first time, the effect of D_2_O on the proliferation of hepatic stellate cells (HSCs). After D_2_O treatment, the p53-cyclin-dependent kinase (CDK) pathway was stimulated, leading to inhibition of the proliferation of HSCs and an increase in the [ATP]/[ADP] ratio. We also evaluated the role of aquaporin (AQP) 11 in activated HSCs. We found that D_2_O treatment decreased AQP11 expression levels. Of note, AQP11 levels elevated by a genetic approach counteracted the D_2_O-mediated inhibition of proliferation. In addition, the expression levels of AQP11 negatively correlated with those of p53. On the other hand, cells transfected with an AQP11-targeted small interfering RNA (siRNA) showed enhanced inhibition of proliferation. These findings suggest that the inhibition of cell proliferation by D_2_O in activated HSCs could be AQP11 dependent. Our previous studies have documented that bisdemethoxycurcumin (BDMC) induces apoptosis by regulating heme oxygenase (HO)-1 protein expression in activated HSCs. In the current study, we tested whether cotreatment with BDMC and D_2_O can modulate the AQP11-dependent inhibition of cell proliferation effectively. We observed that D_2_O cotreatment with BDMC significantly decreased cell proliferation compared to treatment with D_2_O alone, and this effect was accompanied by downregulation of HO-1 and an increase in p53 levels.

## 1. Introduction

Liver fibrosis is a common consequence of chronic liver damage caused by viral infection, oxidative stress, or ethanol consumption [1,2]. Hepatic stellate cells (HSCs), which are a major source of the extracellular matrix, undergo a sequential process of transactivation causing the development of a myofibroblastic phenotype [1,2,3]. This phenotype is associated with increased proliferation and collagen synthesis during fibrogenesis [1,3,4,5]. The excess accumulation of extracellular matrix proteins by activated HSCs inhibits hepatocyte proliferation and induces liver fibrosis, increasing the risk of hepatocellular carcinoma [1,3,4,5]. In the past, liver fibrosis has been considered an irreversible process. Nonetheless, on the basis of the recent paradigm shift in hepatic pathophysiology, researchers now consider hepatic fibrosis a reversible process that takes place via a combination of growth inhibition of activated HSCs and careful control of liver injury [6]. Although the major function of HSCs is the storage of vitamin A, HSCs undergo activation and trans-differentiation to myofibroblasts upon liver damage, thereby causing liver fibrosis [7]. Thus, apoptosis of HSCs may contribute to the termination of fibrogenesis, during the resolution of fibrosis [8].

Aquaporins (AQPs) are a family of membrane proteins that facilitate the transport of water and small solutes [9,10]. The family is composed of 13 isoforms that are distributed widely in nature, from bacteria to animals [9,10]. Of these, mRNA expression of AQPs 0, 1, 5, 8, 9, 11, and 12 has been specifically detected in quiescent HSCs [11]. Of note, the AQP11 protein is clearly expressed in activated HSCs, but the other isoforms are not [11]. Recent studies showed that AQPs are important for cell proliferation [12]. In addition, AQPs are likely to be involved in canalicular and ductal bile secretion, gluconeogenesis, and microbial infections, and may have other novel roles that affect liver function [13]. D_2_O (heavy water) is an analogue of water in which the two hydrogen atoms have been replaced with deuterium [14]. It has been reported that incubation of tumor cells with heavy water leads to inhibition of cell proliferation, thus causing cell cycle arrest, and furthermore, the synergistic effects of D_2_O and chemical agents have been studied before [15,16,17,18]. Our previous studies have been conducted to identify the pharmacological action of phytochemicals that may be able to kill activated HSCs and thereby contribute to the resolution of liver fibrosis [8,19]. In the course of screening of natural products, we have found that bisdemethoxycurcumin (BDMC) can induce cell death in HSCs. In the present study, our purpose was to investigate the effects of D_2_O in combination with BDMC, which has been used as a potent anti-liver fibrosis agent [19], on activated HSCs. To test whether AQP11 plays an important part in HSC proliferation, we treated HSCs with D_2_O. We also focused on the role of AQP11 in activated HSCs [11,20] during the combined effects of D_2_O and BDMC. The present study first examined the effect of D_2_O on proliferation of HSCs. Next, the function of AQP11 in activated HSCs was assessed. Our main purpose was to determine whether the proliferation of activated HSCs is AQP11 dependent under the combined influence of D_2_O and BDMC.

## 2. Results

### 2.1. Activation of the p53-Cyclin-Dependent Pathway by D_2_O Leads to Cell Cycle Arrest

**Figure 1:**
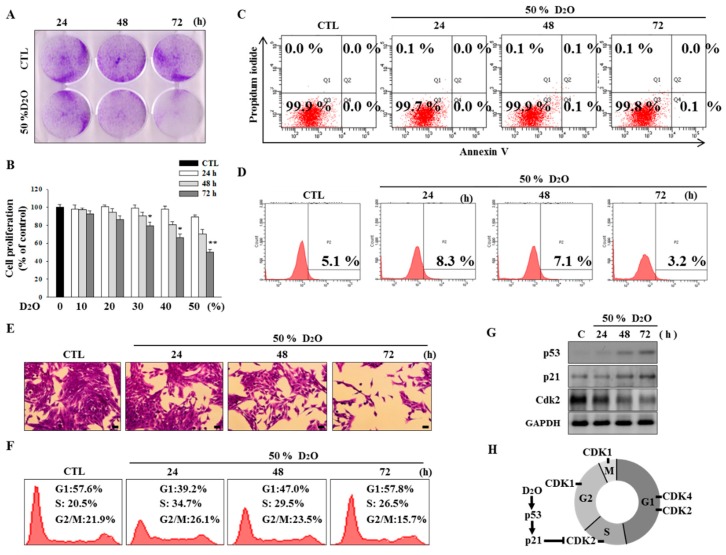
Deuterium oxide (D_2_O) inhibited the proliferation of activated hepatic stellate cells (HSCs). HSC-T6 cells were treated with 50% D_2_O for 24, 48, and 72 h (**A**). The cells were then stained with 0.5% crystal violet and were visualized by photography (**B**). The stained cells were rinsed with dimethyl sulfoxide (DMSO), and absorbance at 570 nm due to the crystal violet staining was measured. The D_2_O-treated HSC cells were stained with Annexin V and propidium iodide (PI), and flow cytometry was performed to determine the degree of apoptosis (**C**). The lower right quadrant (annexin V^+^/propidium iodide [PI]^−^) and upper right quadrant (annexin V^+^/PI^+^) represent the percentage of cells in early and late apoptosis, respectively. To measure intracellular reactive oxygen species (ROS) levels, dichlorodihydrofluorescein diacetate (DCFDA; 20 µM) was added after harvesting of HSCs exposed to 50% D_2_O (**D**). The morphology of stained HSCs was examined by microscopy (scale bar, 100 µm) (**E**). A flow-cytometric analysis of the cell cycle using PI was performed (**F**). The expression levels of p53, p21, and CDK2 were evaluated by western blotting (**G**). GAPDH served as a loading control. A proposed scheme illustrating the mechanism by which D_2_O arrests cells in the S phase via downregulation of aquaporin (AQP) 11 (**H**). ** *p* < 0.01 or * *p* < 0.05 as compared with the control. Data are presented as mean ± SD (n = 3).

The deuterium bonds in D_2_O are stronger than the analogous hydrogen bonds in H_2_O; this phenomenon results in physical differences such as density, viscosity, melting point, and boiling point [21]. Treatment of HSCs with D_2_O inhibited their proliferation in a time- and dose-dependent manner (Figure 1A,B). To elucidate this effect further, we analyzed the apoptotic process and measured reactive oxygen species (ROS) levels. Neither of these processes was affected by D_2_O treatment (Figure 1C,D). Although D_2_O inhibited cell proliferation, we did not observe any sign of cell death (Figure 1E). Subsequently, we evaluated the cell cycle by fluorescence-activated cells sorting (FACS) analysis. The results showed that the percentage of cells in the S phase increased at 24 h, with a gradual decrease thereafter (Figure 1F). As p53, p21, and cyclin-dependent kinases (CDKs) are widely employed to monitor progression of the cell cycle, the expression of these proteins was analyzed in D_2_O-treated HSCs [22]. After D_2_O treatment, we observed upregulation of both p53 and p21 but a reduction in CDK2 protein levels (Figure 1G). This result indicated that D_2_O triggered cell cycle arrest via the p53-CDK pathway. Thus, D_2_O prevented DNA synthesis by activating the S checkpoint, leading to inhibition of HSC proliferation (Figure 1H).

### 2.2. HSC Proliferation Is AQP11 Dependent

**Figure 2:**
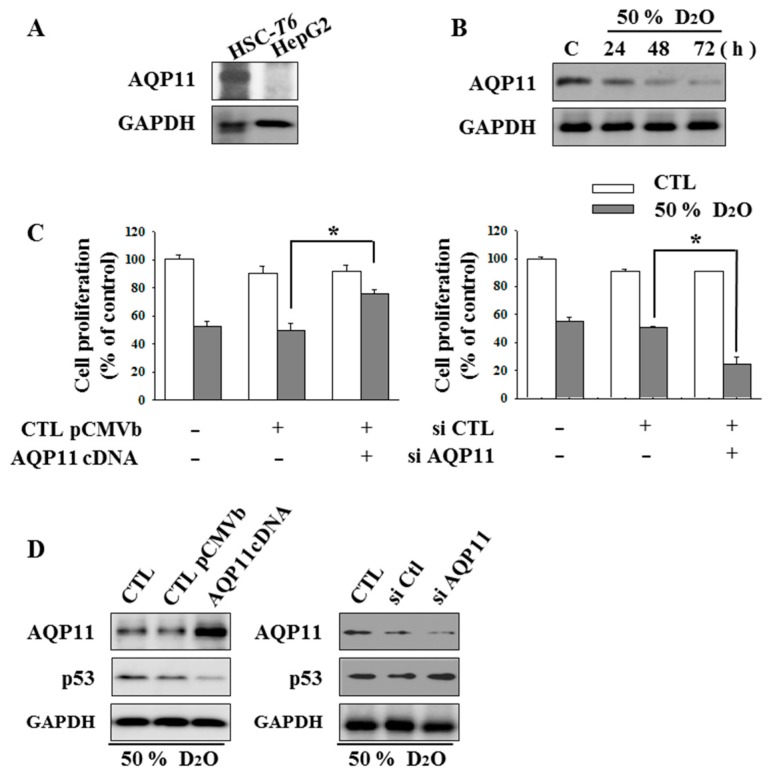
Downregulation of aquaporin (AQP) 11 by deuterium oxide (D_2_O) had an antiproliferative effect on activated hepatic stellate cells (HSCs). AQP11 is expressed in HSC-T6 cells, but not HepG2 cells, as assessed by western blotting (**A**). Intracellular levels of AQP11 were measured by western blotting of lysates from HSC-T6 cells treated with 50% D_2_O for 24, 48, or 72 h (**B**). HSC-T6 cells were transfected with a plasmid containing the AQP11 cDNA or an AQP11-targeted siRNA and then incubated with 50% D_2_O, after which the cells were dyed with crystal violet, and absorbance at 570 nm was quantified to assess cell proliferation (**C**). The expression of AQP11 and p53 was assessed by western blotting. GAPDH served as a loading control (**D**). Data are representative of three independent experiments and are expressed as mean ± SD, * *p* < 0.05.

To gain insights into the role of AQP11 in D_2_O-treated HSCs, we first analyzed the expression of AQP11 in both HSCs and parenchymal HepG2 cells [23]. AQP11 was specifically expressed in HSC-T6 cells (Figure 2A), and D_2_O treatment decreased AQP11 expression levels (Figure 2B). Next, to verify whether AQP11 expression regulates cell proliferation, AQP11 was overexpressed by a genetic approach. Of note, elevated AQP11 levels counteracted the D_2_O-mediated inhibition of proliferation. On the other hand, cells transfected with an AQP11-targeted small interfering RNA (siRNA) showed enhanced inhibition of proliferation (Figure 2C). In addition, the expression levels of AQP11 negatively correlated with those of p53 (Figure 2D).

### 2.3. Inhibition of HO-1 Activity Increases the Antifibrotic Effect of D_2_O

**Figure 3:**
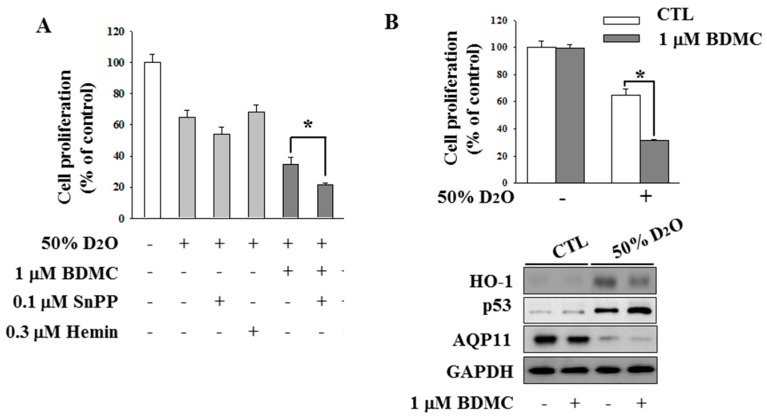
Cotreatment of HSC-T6 cells with deuterium oxide (D_2_O) and bisdemethoxycurcumin (BDMC) decreased the expression of heme oxygenase (HO)-1. HSC-T6 cells were incubated with or without 50% D_2_O for 24 h. In addition, the D_2_O-treated cells were exposed to 1 µM BDMC, 0.1 µM SnPP (tin protoporphyrin), or 0.3 µM hemin (**A**). The effects of cotreatment of HSC-T6 cells with D_2_O and BDMC on their proliferation and expression of HO-1, p53, and AQP11 (**B**). The expression of HO-1, p53, and aquaporin (AQP) 11 was assessed by western blotting. GAPDH served as the loading control. Data are representative of three independent experiments and are expressed as mean ± SD, * *p* < 0.05.

To validate the participation of heme oxygenase (HO)-1 in our experimental setting, we regulated the expression or activity of HO-1 by treating cells with either hemin or SnPP (tin protoporphyrin), respectively. As a result, we observed a reduction in proliferation of HO-1–deficient cells, as compared to a control (Figure 3). Our previous study has shown that BDMC, a natural derivative of curcumin, induces apoptosis selectively in activated HSCs [19]. In the present study, we cotreated cells with D_2_O and a low concentration of BDMC. D_2_O cotreatment with BDMC significantly decreased cell proliferation compared to treatment with D_2_O alone (Figure 3A), and this effect was accompanied by downregulation of HO-1 and an increase in p53 levels (Figure 3B).

### 2.4. Excess Accumulation of ATP Diminishes Cell Proliferation

**Figure 4:**
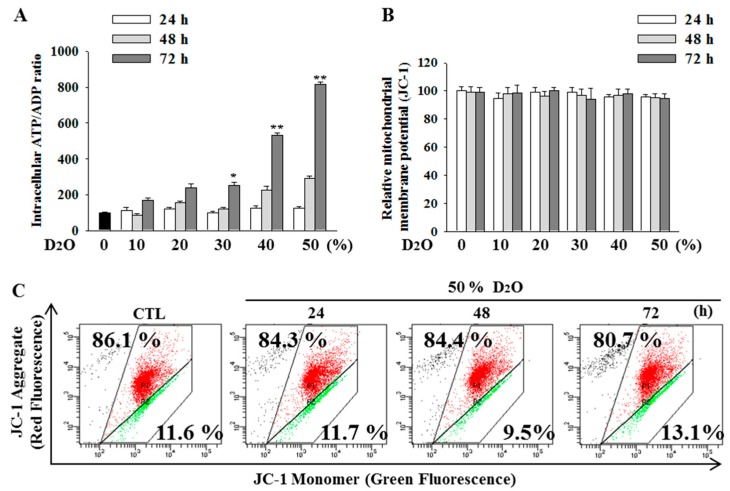
The [ATP]/[ADP] ratio increased after treatment of HSC-T6 cells with deuterium oxide (D_2_O) for 24, 48, or 72 h. ATP and ADP levels were measured and expressed as the [ATP]/[ADP] ratio (**A**). Mitochondrial membrane potential was measured with the JC-1 dye (**B**,**C**). ** *p* < 0.01 or * *p* < 0.05 as compared with the control. Data are presented as mean ± SD (n = 3).

The balance between the synthesis and turnover of ATP may be affected in cells treated with D_2_O. D_2_O increased the ratio [ATP]/[ADP] in a time- and dose-dependent manner (Figure 4A). To test whether the changes in ATP levels were accompanied with augmentation of mitochondrial dysfunction [24], the [ATP]/[ADP] ratio was measured by JC-1 staining. Nonetheless, there were no noticeable changes in mitochondrial membrane potential following D_2_O treatment (Figure 4B,C).

## 3. Discussion

Recently, it was suggested that AQPs are involved in the regulation of cell proliferation [25], but the exact mechanism of action of AQP11 is unclear. AQP3 is a typical aquaglyceroporin that is capable of transporting water, glycerol, and urea, and therefore plays a major role in fluid homeostasis in normal tissues [26]. A recent paper revealed that an AQP3 knockdown reduces cellular glycerol content and mitochondrial ATP formation in A549 and H1299 cells [26]. In line with the previous reports, we focused on the function of AQP11 in activated HSCs. We hypothesized that AQP11-facilitated glycerol transport is an important determinant of HSC proliferation through a mechanism in which cellular glycerol is the key regulator of cellular ATP levels. To determine whether AQP11 plays an important part in HSC proliferation, we treated HSCs with D_2_O. As a result, D_2_O inhibited HSC proliferation through activation of the p53-CDK pathway (Figure 1) and decreased the AQP11 amount in HSCs. Furthermore, we observed upregulation of the [ATP]/[ADP] ratio (Figure 4). Moreover, the antifibrotic effect of D_2_O was found to be AQP11 dependent (Figure 2). AQP11 was specifically expressed in HSC-T6 cells (not in HepG2 cells; Figure 2A). Via a genetic approach, we overexpressed AQP11 and confirmed that the elevated AQP11 levels counteracted the D_2_O-mediated inhibition of proliferation; on the other hand, cells transfected with the AQP11-targeted siRNA manifested greater inhibition of proliferation during D_2_O treatment (Figure 2C). Furthermore, the expression levels of AQP11 negatively correlated with those of p53, which can regulate cell proliferation (Figure 2D). These results suggest that the inhibition of cell proliferation by D_2_O in activated HSCs could be AQP11 dependent.

Given that HSC proliferation is heme oxygenase (HO) dependent [19], we next examined the expression of HO-1 in activated HSCs treated with D_2_O. It is generally accepted that upregulation of HO-1 is cytoprotective in activated HSCs [19]. Thus, inhibition of HO-1 expression may attenuate HSC proliferation. This finding paradoxically indicates that the protective activity of HO-1 seems to be a double-edged sword because inhibition of HSC proliferation attenuates rat liver fibrosis [27]. Our previous studies have documented that BDMC induces apoptosis by regulating HO-1 protein expression in activated HSCs [19]. In the present study, we tested whether cotreatment with BDMC and D_2_O can modulate the AQP11-dependent inhibition of cell proliferation effectively [28]. Our data revealed that BDMC coadministered with D_2_O markedly decreased cell proliferation by downregulating some cellular proteins including HO-1 and AQP11 and by inducing p53 (Figure 3).

Additionally, we observed an increase in the [ATP]/[ADP] ratio in HSCs treated with D_2_O, although mitochondrial membrane potential and membrane integrity remained almost unchanged. One important cellular function of H_2_O is participation in the conversion of ATP to ADP by hydrolysis. This fact suggests that the decreased rate of ATP hydrolysis may not produce enough protons in a living cell, and this phenomenon may partially be responsible for the inhibition of proliferation [29]. In support of this finding, D_2_O has been reported to decrease ATPase activity and ATP-hydrolytic activity in maize root segments in vivo [30,31]. Our experiments revealed that D_2_O increased the [ATP]/[ADP] ratio in a time- and dose-dependent manner (Figure 4) but did not affect mitochondrial membrane potential. These findings suggest that D_2_O shifted the balance in activated HSCs toward antiproliferative effects. Furthermore, it has been reported that upregulation of HO-1 enhances renal mitochondrial transporter carriers [32]. Thus, elevated [ATP]/[ATP] may partially be mediated by HO-1 activity. Therefore, a further study will be required to discover a direct relation between the [ATP]/[ATP] ratio and HO-1 in the cell treated with D_2_O.

Therefore, in this study, we focused on the role of AQP11 by investigating its expression in activated HSCs during D_2_O treatment. In addition, because D_2_O has been reported to be active toward various cell lines in vitro in combination with phytochemicals, we evaluated, for the first time, the effect of D_2_O with a phytochemical on proliferation of HSCs. Our results suggest that D_2_O may contribute to the inhibition of proliferation by downregulating AQP11 in activated HSCs.

## 4. Materials and Methods

### 4.1. Cell and Tissue Cultures

The immortalized rat hepatic stellate cell line HSC-T6 was provided by Prof. S.H. Sung (Seoul National University, Seoul, Korea). The immortalized HSC-T6 cells retain virtually all the features of activated HSCs. The human liver carcinoma cell line (HepG2) was supplied by the Korean Cell Line Bank (Seoul, Korea). HSCs and HepG2 cells were cultured in Dulbecco’s modified Eagle’s medium (DMEM) supplemented with 10% of fetal bovine serum (FBS) and 1% of a penicillin and streptomycin solution (Pen Strep) in a humidified atmosphere containing 5% of CO_2_ at 37 °C, according to a protocol supplied by the Korean Cell Line Bank.

### 4.2. Reagents and Chemicals

D_2_O and BDMC were purchased from the Sigma-Aldrich Corporation (St. Louis, MO, USA).

### 4.3. Cell Proliferation

This parameter was measured by an established crystal violet assay. HSC-T6 cells were seeded in a dish with a 60 mm diameter at a density of 2 × 10^4^ cells/cm^2^ and grown at 37 °C overnight. The water in the culture medium was replaced with 50% D_2_O, and the incubation was continued for 24, 48, and 72 h. The cells were washed twice with phosphate-buffered saline (PBS), fixed with methanol for 15 min, and stained with 0.5% crystal violet for 15 min at room temperature. Stained cells were then dissolved in 1 mL of dimethyl sulfoxide (DMSO), and absorbance was measured at 570 nm.

### 4.4. An Apoptosis Assay

Cells were incubated for various periods with 50% D_2_O. The cells were detached with ethylenediaminetetraacetic acid (EDTA)-free trypsin and washed twice with cooled PBS. Then, the cells were resuspended in 400 µL of 1× loading buffer containing 5 µL annexin V and 5 µL propidium iodide (PI; Becton-Dickinson, San Diego, CA, USA) for 15 min on ice in the dark. The assays of apoptosis were performed on a FACS Calibur analyzer (Becton-Dickinson, San Diego, CA, USA).

### 4.5. Western Blotting and Antibodies

Cells were lysed with radioimmunoprecipitation (RIPA) assay buffer consisting of 1× PBS, 1% (*v*/*v*) Nonidet P-40 (NP-40), 0.5% (*w*/*v*) sodium deoxycholate, 0.1% (*w*/*v*) sodium dodecyl sulfate (SDS), 0.1 mg/mL phenylmethylsulfonyl fluoride (PMSF), 30 μL/mL aprotinin, and 1 mM sodium orthovanadate (Na_2_VO_3_). The cell lysates were centrifuged, and the resulting supernatants were collected. Proteins were separated by SDS-PAGE on 8–15% gels and then transferred to a polyvinylidene difluoride (PVDF) membrane. Each membrane was blocked in Tris-buffered saline containing 0.1% of Tween 20 (TBST) and 5% nonfat dry milk for 1 h at room temperature, and then incubated overnight with primary antibodies in TBST containing 1% nonfat dry milk at 4 °C. Anti-cyclin-dependent kinase 2 (CDK2), anti-p53, anti-p21, anti-AQP11, anti-heme oxygenase (HO)-1, and anti-glyceraldehyde phosphate dehydrogenase (GAPDH) antibodies were purchased from Santa Cruz Biotechnology (Dallas, TX, USA). The membranes were washed with TBST and incubated with a horseradish peroxidase–conjugated secondary antibody: either a goat anti-rabbit IgG or goat anti-mouse IgG antibody for 2 h. Signals were detected using a chemiluminescence system (GE Healthcare, Piscataway, NJ, USA).

### 4.6. The Intracellular ATP/ADP Ratio

ATP and ADP concentrations were measured by means of ATP Bioluminescence Assay Kit HS II (Roche Applied Science, Monza, Italy) and the ADP Colorimetric/Fluorometric Assay Kit (Sigma-Aldrich). Cells were harvested, counted, and lysed with the above-mentioned lysis buffer. Aliquots (50 µL) from each diluted sample or standard were transferred to 96-well plates, after which 50 µL of a luciferase reagent was added. After mixing, the emitted light was measured and integrated over 10 s on an automated microplate luminometer (Bio-Tek, San Diego, CA, USA).

### 4.7. Analysis of the Cell Cycle

Hepatic stellate cells T6 (3 × 10^5^ cells/well) were dispensed into 6-well plates and treated with D_2_O for 24, 48, and 72 h. The cells were harvested and fixed in 70% ethanol and then stored at −20 °C for 24 h. The cells were washed twice with PBS and resuspended in 100 µL of RNase A at 37 °C, followed by staining with 50 µg/mL PI for 10 min at room temperature in the dark. The stained cells were quantified on a flow cytometer: FACS Calibur analyzer (Becton-Dickinson, San Diego, CA, USA). DNA content in the G0 (or G1) phase, S phase, and at the G2–M transition was analyzed in the BD Calibur software (version 1.0.264.21; BD Biosciences, San Jose, CA, USA). The cell cycle consists of four distinct phases: The G1 phase, S phase (synthesis), G2 phase (collectively known as interphase), and M phase (mitosis or meiosis). The M phase is itself composed of two tightly coupled processes: karyokinesis, during which the cell’s chromosomes divide, and cytokinesis, where the cell’s cytoplasm divides forming two daughter cells. Activation of each phase is dependent on the proper progression and completion of the previous one. Cells that have temporarily or reversibly stopped dividing are said to have entered a state of quiescence called the G0 phase.

### 4.8. Intracellular ROS Levels

The intracellular production of ROS was analyzed with a fluorescent probe: DCFDA. Cells were incubated with 50% D_2_O for 24, 48, and 72 h prior to harvesting. After all the HSC-T6 cells were collected by centrifugation, the supernatant was removed. Each pellet was resuspended in PBS, and 20 μM DCFDA was added. Fluorescence was measured by flow cytometry.

### 4.9. Statistical Analysis

All the experiments were conducted at least in triplicate, and data were expressed as mean ± standard deviation. Statistical significance was determined by the *t* test or one-way analysis of variance (ANOVA) with Tukey’s test at indicated *p* values in the SPSS 18.0 software (SPSS Inc., Chicago, IL, USA).
